# Influence of Screen Time during COVID-19 on Health-Related Quality of Life of Early Adolescents

**DOI:** 10.3390/ijerph191710498

**Published:** 2022-08-23

**Authors:** Mei-Chun Cheung, Joanne Yip, Jason Pui Yin Cheung

**Affiliations:** 1 Department of Social Work, The Chinese University of Hong Kong, Shatin, New Territories, Hong Kong SAR, China; 2 School of Fashion and Textiles, The Hong Kong Polytechnic University, Hung Hom, Kowloon, Hong Kong SAR, China; 3 Department of Orthopaedics and Traumatology, The University of Hong Kong, Hong Kong SAR, China

**Keywords:** screen time, health-related quality of life, early adolescents

## Abstract

This study investigated the influence of screen time during COVID-19 on the physical and mental domains of the health-related quality of life of early adolescents. A total of 860 early adolescents were recruited. The 36-Item Short Form Health Survey was used to measure their health-related quality of life. The early adolescents reported their average daily time spent using smartphones and computers and watching television over the previous week. The results show that most early adolescents, on average, spent less than 1 h to more than 4 h per day during COVID-19 using smartphones (*n* = 833, 96.9%) and computers (*n* = 783, 91.0%), and watching television (*n* = 804, 93.5%), respectively. Though early male and female adolescents spent a similar amount of time daily on average using smartphones, early male adolescents spent more time using computers and watching television than early female adolescents and reported a significantly lower mean score for three out of the eight scales in the physical and mental domains of health-related quality of life. While health-related quality of life of early female adolescents was negatively associated with time spent using smartphones only, early male adolescents were adversely affected by the time spent using smartphones and computers and watching television (*p* < 0.05). Therefore, early adolescents who spent more time using display devices during COVID-19 had significantly poorer outcomes in their health-related quality of life, and gender difference was found in the influence of screen time on health-related quality of life.

## 1. Introduction

The coronavirus disease 2019 (COVID-19) pandemic has had a significant impact on the lives of children and adolescents around the world. Due to the lockdowns or school closures, many children and adolescents have stopped going to school and stayed at home as a public health measure to reduce the transmission of COVID-19 in the community and protect them from infection. In response to school closures, primary and secondary schools have gradually transitioned to virtual or online curriculums for students to maintain their learning and engage in school-related activities. As a result, children and adolescents rapidly switched from face-to-face to online schooling using display devices, such as computers and smartphones, and spent more time in front of display devices. Studies conducted in various countries revealed that a sharp increase in screen time for children and adolescents was associated with the first wave of COVID-19 worldwide in 2020 [1,2,3,4,5,6,7,8]. A survey of Canadian parents carried out during the height of the COVID-19 lockdown between June and August 2020 found that children’s time spent watching content on screens and playing video games substantially increased, from 2.6 h during the pre-pandemic period to 5.9 h per day during the pandemic. In total, media screen time increased by over 3 h per day with the onset of the pandemic [9]. Therefore, the use of display devices has increased among early adolescents due to the fundamental need for online education, social contact, and entertainment resulting from school closures and limited access to outdoor activities since the outbreak of COVID-19.

Research conducted before the pandemic consistently demonstrated that more screen time is associated with lower health-related quality of life [10,11,12]. Specifically, more than two hours of screen time per day was significantly associated with various adverse health outcomes and lower quality of life. Excessive screen time is also associated with negative consequences regarding children’s physical, cognitive, and mental health [13]. Our previous study conducted before the pandemic [14] also suggested that early adolescents who spent more time using smartphones and computers have significantly poorer outcomes in the physical and mental domains of their health-related quality of life. Children and adolescents can use screens to keep socially connected with others during the lockdown or school closures by engaging in various online school-related or recreational activities and receive health educational resources and support services via online platforms [15,16]. However, recent studies during COVID-19 demonstrated that children with higher screen use levels had significantly higher mental health symptoms [4,17] and poor lifestyle behavior [18].

Like many other countries worldwide, schools closed down rapidly in Hong Kong after the announcement of the COVID-19 outbreak and the identification of confirmed cases in early 2020. Initially, all schools in Hong Kong were suspended for 4 weeks, starting from 1 February 2020. With more stringent social distancing measures implemented, school closures were extended to April 2020 [19]. As the use of display devices is inevitable during school closure or social distancing in response to COVID-19 in Hong Kong, the increase in screen time during the ongoing pandemic is likely to create a greater risk of adverse health outcomes in early adolescents. Therefore, it is more important than ever to explore whether the use of display devices during COVID-19 displaces social activities, leading to social withdrawal and a decline in well-being (in line with the displacing social activity hypothesis), or enables the development of additional social connections established on the internet (in line with the displacing strong ties hypothesis), as proposed by Kraut et al. [20]. To bridge this research gap, this study explored the use of display devices among early adolescents in Hong Kong during COVID-19 and the influence of different display devices on their health-related quality of life. The preliminary results in our previous study based on disproportionate sample sizes of early male and female adolescents indicated no significant difference in the average daily time spent using a smartphone between males and females. In contrast, early male adolescents spent more time on computers than early female adolescents [14]. As the sample size of early male adolescents was relatively small (*n* = 81), these preliminary findings may not be conclusive. To substantiate any gender difference in terms of the time spent using display devices and their differential influence on health-related quality of life, this study built on our previous study with comparable male and female samples and larger sample sizes. More specifically, this study aimed to investigate (1) the amount of time spent using different display devices, including smartphones, computers, and televisions, among early adolescents of both genders in Hong Kong and (2) their influences on the physical and mental domains of health-related quality of life of early adolescents. Based on our previous study [14] and other studies [1,2,3,4,5,6,7,8,9], it was hypothesized that an increase in screen time would be observed in early adolescents in Hong Kong during the pandemic. Moreover, early male adolescents spent more time using display devices and had poorer health-related quality of life than early female adolescents. In general, early adolescents who spent more time using display devices during COVID-19 had significantly poorer outcomes in their health-related quality of life, in line with the displacing social activity hypothesis proposed by Kraut et al. [20].

## 2. Materials and Methods

### 2.1. Participants

A cross-sectional study using self-report questionnaires was conducted during COVID-19 from 2020 to 2021 in Hong Kong. Through convenience sampling, 860 early adolescents (male = 430, female = 430) between 9 and 14 years old were recruited from various primary and secondary schools through two channels in Hong Kong. The first channel was direct recruitment through their primary and secondary schools during weekends when the schools were still permitted to offer extra-curricular activities during 0which the pandemic was relatively under control in the community. This channel recruited a total of 484 early adolescents. When the schools were entirely suspended for schooling due to different surging waves of COVID-19, the remaining adolescents (*n* = 376) were recruited through the second channel via open recruitment, which was advertised through online social platforms and carried out at The Hong Kong Polytechnic University with appropriate social distancing measures being executed in accordance with the guidelines set by the government. Upper body posture screening was provided to the early adolescents as an incentive. Early adolescents filled in the self-report questionnaires anonymously and independently within sight of their parents or guardians, who sat in the waiting area located nearby. After completion, the early adolescents submitted their self-report questionnaires to the research assistants before undergoing posture screening. The questionnaires were double-checked by the research assistants to avoid multiple submissions.

All early adolescents took part voluntarily, and informed assent and written informed consent for participation were obtained from early adolescents and their parents or guardians before the commencement of the study. The study was conducted according to the guidelines of the Declaration of Helsinki, and the research protocol was approved by the Survey and Behavioural Research Ethics Committee and the Joint Chinese University of Hong Kong—New Territories East Cluster Clinical Research Ethics Committee of The Chinese University of Hong Kong. All methods were performed following the relevant guidelines and regulations. The demographic data and descriptive statistics on the main variables of interest in early adolescents are provided in Table 1.

### 2.2. Measures

#### 2.2.1. Health-Related Quality of Life

Early adolescents’ health-related quality of life in the physical and mental domains was measured using the Hong Kong version [21,22,23,24] of the 36-item Short Form Health Survey (SF-36) [25,26], which has been adapted and validated for over 40 populations [27] and is commonly used in adolescents [28,29,30], with norm references available from 14 populations [27], including Hong Kong [21,22]. The SF-36 consists of 35 questions that are summarized into eight multi-item scales under two domains and 1 self-reported question related to changes in health compared to one year ago. The four scales related to the physical domain of health-related quality of life are physical functioning (10 items), role limitation due to physical health problems (4 items), bodily pain (2 items), and general health (5 items), whereas the four scales related to the mental domain of health-related quality of life are vitality (energy/fatigue; 4 items), social functioning (2 items), role limitation due to emotional problems (3 items), and mental health (5 items) [31]. The items in each scale are summed and transformed according to the standard scoring algorithm of the SF-36 [26] into a standardized scale score for each scale that ranges from 0 to 100, with a higher scale score indicating better health-related quality of life. As this study does not focus on changes in health over time, the self-reported health transition item, an independent question not used to score any of the eight multi-item scales [26], was not used for analysis in this study.

#### 2.2.2. Smartphone, Computer, and Television Use

A custom questionnaire was used to measure the time that early adolescents spent using smartphones and computers and watching television. The respondents were asked to rate their average daily time spent using smartphones and computers and watching television over the previous week on the following scale: (a) never, (b) less than one hour, (c) one to two hours, (d) three to four hours and (e) more than four hours. The scales for the average daily time spent performing these activities were adapted from the study by Kratěnová et al. [32], where it was determined that early adolescents spent an average of 2 h per day using the computer. The questionnaire has been used in our previous studies [14,33], where the scales generally achieved a normal distribution for the collected data.

### 2.3. Data Processing and Analysis

All the statistical analyses were performed using the Statistical Package for Social Sciences (SPSS, Version 25.0, IBM Corp, Armonk, NY, USA). Descriptive statistics were generated for the total sample and each gender sub-sample. Quantitative measurements are summarized as the mean ± standard deviation and *n* (%). The comparison of the amount of time spent by early adolescents using smartphones and computers and watching television was performed with a paired-sample t-test, and comparisons between early male and female adolescents on the amount of time spent using smartphones, computers, and watching television were performed through independent sample t-tests followed by nonparametric Mann–Whitney U tests. Bivariate Pearson’s correlations were used to measure the relationship between the average daily time spent using smartphones and computers and watching television and the physical and mental domains of health-related quality of life. Stepwise multiple linear regressions were conducted to assess whether the average daily amount of time spent by early adolescents using smartphones and computers and watching television was a significant predictor of each of the eight scales related to the physical and mental domains of health-related quality of life for early male and female adolescents, respectively. The prevalence of smartphone and computer use was also compared to our previous study, which was conducted before COVID-19 [14], to assess whether there was any increase in screen time during the pandemic. Preliminary analyses were conducted to ensure no violation of the assumptions of normality, linearity, multicollinearity, or homoscedasticity. A *p*-value of less than 0.05 was considered to be statistically significant.

## 3. Results

### 3.1. Gender Difference in the Prevalence of Smartphone, Computer and Television Use during COVID-19

The result of the paired-sample t-test suggested that during COVID-19, early adolescents overall had spent a similar amount of time using smartphones and computers daily on average over the previous week, *t*(859) = 0.094, *p* > 0.05. However, they spent significantly less time watching television, as compared to using both smartphones, *t*(859) = −7.072, *p* < 0.001, and computers, *t*(859) = −8.183, *p* < 0.001. Similar findings were observed when the analyses were carried out in the male and female sub-samples.

**Figure 1:**
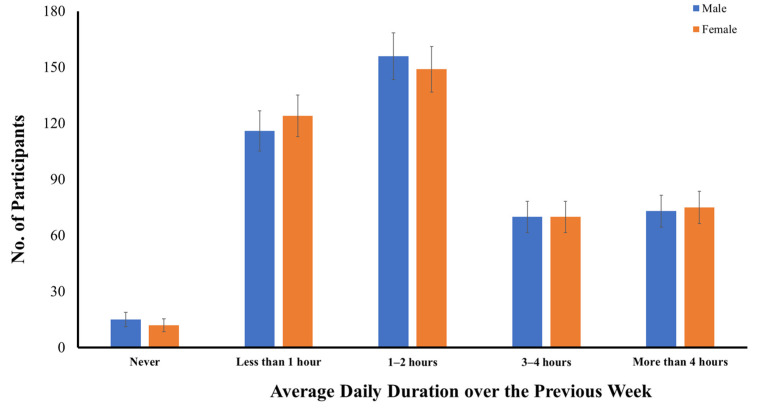
Distribution of early adolescents categorized across gender based on their average daily time spent using a smartphone over the previous week.

Figure 1 shows the numbers of early adolescents categorized across gender based on their average daily time spent using a smartphone over the previous week during COVID-19. While 15 male and 12 female adolescents reported no smartphone usage over the previous week, most early adolescents (*n* = 833, 96.9%) were smartphone users. No significant difference between early male and female adolescents was found in their average daily time spent using a smartphone over the previous week, in either the parametric, *t*(1, 858) = −0.061, *p* > 0.05, or the nonparametric test, *U* = 92,335.5, z = −0.033, *p* > 0.05. On average, 27.9% of the early adolescents used their smartphones for less than one hour, 35.5% used their smartphones for one to two hours per day, 16.3% used their smartphones for three to four hours per day, and 17.2% used their smartphones for more than four hours per day during COVID-19.

**Figure 2:**
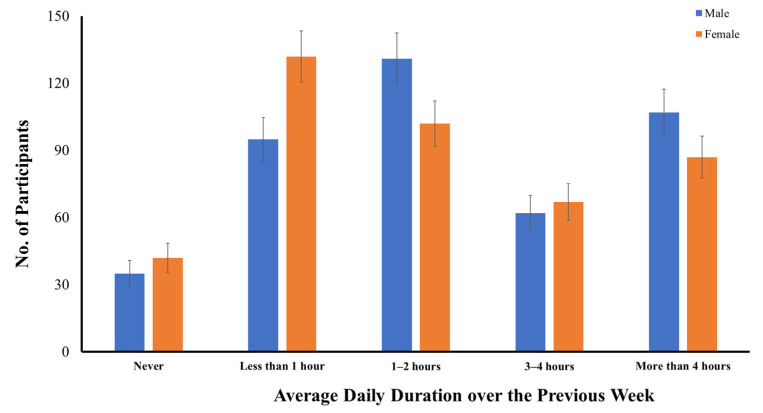
Distribution of early adolescents categorized across gender based on their average daily time spent using a computer over the previous week.

As compared to smartphone use, more male (*n* = 35) and female (*n* = 42) adolescents reported no computer use over the previous week, and only 91.0% (*n* = 783) were computer users during COVID-19 (Figure 2). On average, 26.4% of early adolescents used their computers for less than one hour, 27.1% used their computers for one to two hours per day, 15.0% used their computers for three to four hours per day, and 22.6% used their computers for more than four hours per day. Unlike smartphone use, there was a significant difference between early male and female adolescents in their average daily time spent using a computer over the previous week, in both the parametric, *t*(1, 858) = 2.288, *p* < 0.05, and nonparametric tests, *U* = 83,885.5, *z* = 2.417, *p* < 0.05. In general, early male adolescents had spent more time on computers than early female adolescents over the previous week. While only 20.2% of early female adolescents (*n* = 87) had used their computers for more than four hours per day, more early male adolescents (*n* = 107, 24.8%) had used their computers for similar hours per day over the previous week.

**Figure 3:**
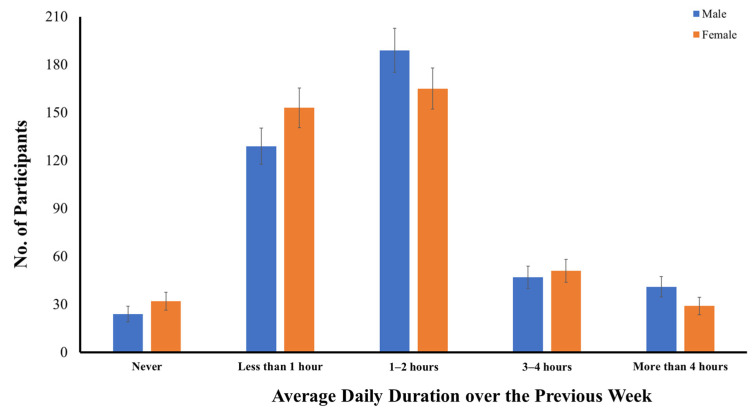
Distribution of early adolescents categorized across gender based on their average daily time spent watching television over the previous week.

Figure 3 shows the numbers of early adolescents categorized across gender based on their average daily time spent watching television over the previous week. A total of 804 early adolescents had watched television over the previous week during COVID-19, accounting for nearly 93.5% of the sample. On average, 32.8% of the early adolescents watched television for less than one hour, 42.2% watched television for one to two hours per day, 11.4% watched television for three to four hours per day, and 8.1% watched television for more than four hours per day. A similar significant gender difference was also found in their average daily time spent watching television, in both the parametric, *t*(1, 858) = 2.054, *p* < 0.05, and nonparametric tests, *U* = 85,278.0, *z* = 2.084, *p* < 0.05. Early male adolescents had spent more time watching television than early female adolescents over the previous week during COVID-19 (Figure 3).

### 3.2. Gender Difference in Health-Related Quality of Life

Descriptive statistics on the scales of SF-36 for early male and female adolescents are provided in Table 1. Compared with early female adolescents, early male adolescents reported a significantly lower mean score for three of the eight scales under the physical and mental domains of health-related quality of life. The results of independent samples t-tests revealed statistically significant differences between the two groups in the physical scales of physical functioning (*M*_diff_ = −1.698, 95% CI [−3.252, −0.144], *t*(858) = 2.144, *p* < 0.05), role limitation due to physical problems (*M*_diff_ = −2.035, 95% CI [−3.929, −0.140,], *t*(858) = −2.108, *p* < 0.05), and in the mental scales of social functioning (*M*_diff_ = −2.006, 95% CI [−3.639, −0.372], *t*(858) = −2.410, *p* < 0.05).

### 3.3. Influences of the Durations of Smartphone, Computer, and Television Use on Early Adolescents’ Health-Related Quality of Life across Gender during COVID-19

Given that the durations of smartphone, computer, and television use were significantly correlated with one another, *p* < 0.001, and a gender difference was found in the average daily time spent using computers and watching television during COVID-19, stepwise multiple linear regressions were conducted separately for early male and female adolescents to predict the associated physical and mental domains of early adolescents’ health-related quality of life based on their average daily durations of smartphone, computer, and television use during COVID-19. The results are presented in Table 2 and Table 3.

#### 3.3.1. Early Male Adolescents

Among early male adolescents, the duration of smartphone use was the first significant predictor entered into the regression models for five scales of health-related quality of life during COVID-19 (Table 2). For the physical domain of health-related quality of life, the duration of smartphone use reached statistical significance in predicting 1.4% of the variance in physical functioning, *F*(1, 428) = 6.038, *p* < 0.05, *R*^2^ = 0.014; 1.2% of the variance in role limitation due to physical problems, *F*(1, 428) = 5.417, *p* < 0.05, *R*^2^ = 0.012; and 1.4% of the variance in bodily pain, *F*(1, 428) = 5.958, *p* < 0.05, *R*^2^ = 0.014. The duration of smartphone use negatively affected physical functioning (*b* = −1.446, *p* < 0.05), role limitation due to physical problems (*b* = −1.677, *p* < 0.05), and bodily pain (*b* = −1.431, *p* < 0.05). For the mental domain of quality of life, the duration of smartphone use also reached statistical significance in predicting 1.6% of the variance in vitality (energy/fatigue), *F*(1, 428) = 6.789, *p* < 0.01, *R*^2^ = 0.016, and 3.4% of the variance in mental health, *F*(1, 428) = 14.860, *p* < 0.001, *R*^2^ = 0.034. The duration of smartphone use negatively affected vitality (energy/fatigue) (*b* = −1.882, *p* < 0.01) and mental health (*b* = −2.633, *p* < 0.001); that is, early male adolescents who spent a longer average time daily on smartphone use had poorer health-related quality of life in terms of physical functioning, role limitation due to physical problems, bodily pain, vitality (energy/fatigue), and mental health.

The duration of computer use significantly predicted one scale related to the physical domain, namely, general health, and one related to the mental domain of health-related quality of life, namely, social functioning. The duration of computer use reached statistical significance in predicting 1.8% of the variance in general health, *F*(1, 428) = 7.784, *p* < 0.01, *R*^2^ = 0.018, and 1.0% of the variance in social functioning, *F*(1, 428) = 4.186, *p* < 0.05, *R*^2^ = 0.010. The duration of computer use negatively affected general health (*b* = −1.783, *p* < 0.01) and social functioning (*b* = −1.013, *p* < 0.05); that is, early male adolescents who spent a longer average time daily on computer use had poorer health-related quality of life in terms of general health and social functioning. After the effects of the average daily amount of time spent by early male adolescents using computers were controlled, the average amount of time spent using a smartphone per day was entered into the regression models and still significantly predicted one scale related to the physical domain of health-related quality of life, namely, general health. The duration of smartphone use statistically significantly predicted an additional 1.0% of the variance in general health, *F*(1, 427) = 4.425, *p* < 0.05, *R*^2^ change = 0.010. As a result, both the duration of smartphone use and the duration of computer use significantly predicted the variance in general health, accounting for a total of 3.6% of the variance in general health vitality, *F*(2, 427) = 6.136, *p* < 0.01, *R*^2^ = 0.028.

The duration of watching television also significantly predicted one scale related to the mental domain of health-related quality of life, namely, role limitation due to emotional problems. The duration of television use reached statistical significance in predicting 1.0% of the variance in role limitation due to emotional problems, *F*(1, 428) = 4.117, *p* < 0.05, *R*^2^ = 0.010. The duration of television use negatively affected role limitation due to emotional problems (*b* = −2.258, *p* < 0.05); that is, early male adolescents who spent a longer duration of watching television on average daily had poorer health-related quality of life in terms of role limitation due to emotional problems.

#### 3.3.2. Early Female Adolescents

Table 3 shows that the duration of smartphone use was the only significant predictor entered into the regression models for seven domains in early female adolescents during COVID-19. For the physical domain of health-related quality of life, the duration of smartphone use reached statistical significance in predicting 1.4% of the variance in physical functioning, F(1, 428) = 6.259, *p* < 0.05, R^2^ = 0.014; 2.4% of the variance in role limitation due to physical problems, F(1, 428) = 10.417, *p* < 0.01, R^2^ = 0.024; and 2.6% of the variance in general health, F(1, 428) = 11.210, *p* < 0.001, R^2^ = 0.026. The duration of smartphone use negatively affected physical functioning (b = −0.996, *p* < 0.05), role limitation due to physical problems (b = −1.547, *p* < 0.01), and general health (b = −2.471, *p* < 0.001). For the mental domain of quality of life, the duration of smartphone use also reached statistical significance in predicting 4.4% of the variance in vitality (energy/fatigue), F(1, 428) = 19.909, *p* < 0.001, R^2^ = 0.044; 1.2% of the variance in social functioning, F(1, 428) = 5.416, *p* < 0.05, R^2^ = 0.012; 1.4% of the variance in role limitation due to emotional problems, F(1, 428) = 6.066, *p* < 0.05, R^2^ = 0.014; and 3.4% of the variance in mental health, F(1, 428) = 15.014, *p* < 0.001, R^2^ = 0.034. The duration of smartphone use negatively affected vitality (energy/fatigue) (b = −3.220, *p* < 0.001), social functioning (b = −1.128, *p* < 0.05), role limitation due to emotional problems (b = −2.186, *p* < 0.05), and mental health (b = −2.553, *p* < 0.001); that is, early female adolescents who spent a longer average time daily on smartphone use had poorer health-related quality of life in terms of physical functioning, role limitation due to physical problems, general health, vitality (energy/fatigue), social functioning, role limitation due to emotional problems, and mental health.

## 4. Discussion

This study aimed to investigate the influence of screen time during COVID-19 on the physical and mental domains of health-related quality of life of early adolescents across gender in Hong Kong. The results revealed that most early adolescents, on average, spent less than 1 h to more than 4 h per day during COVID-19 using smartphones (96.9%) and computers (91.0%) and watching television (93.5%). Regarding the use of different display devices, early adolescents had spent a similar amount of time using smartphones and computers daily on average over the previous week. Still, they had spent less time watching television than using smartphones and computers during the pandemic. With the expansion of broadcast channels through online platforms, it is conceivable that early adolescents can use smartphones and/or computers to watch or stream movies, videos, and/or shows originally accessible by television. In addition, with multiple functions being combined into one device and technology’s integration into the learning experience, smartphones and/or computers have brought convenience and flexibility to early adolescents. Therefore, apart from recreational activities, such as texting, video chatting, browsing the internet, playing games, and social media, they can use smartphones and/or computers for school-related tasks, resulting in more time spent using smartphones and computers and accounting for the variation in the time spent using different types of display devices.

As compared to our previous study conducted before the pandemic [14], the prevalence of smartphone users is relatively similar before (98.8%) and during COVID-19 (96.9%). However, the prevalence of computer users dramatically increased from 74.9% before COVID-19 to 91.0% during COVID-19, suggesting that more early adolescents used computers during COVID-19. In addition, the percentages of early adolescents spending an average of 3 h or more daily using smartphones (before COVID-19—24.9%; during COVID-19—33.5%) and computers (before COVID-19—7.08%; during COVID-19—37.6%) also significantly increased during the pandemic. In line with our findings, several studies have reported that increased screen time is commonly observed in children worldwide during the pandemic [1,2,3,4,5,6,7,8,9]. As activities outside the home were prohibited due to lockdown or school closure during COVID-19, early adolescents undoubtedly spent more time in front of display devices for online learning and recreational activities as part of their activities at home.

Given the discrepancy in the number of male and female adolescents in the sample of our previous study [14], this study addressed this limitation by recruiting more male adolescents to explore gender effects on the use of display devices and their influence on health-related quality of life across gender. While early male and female adolescents spent similar amounts of time using smartphones, a gender difference was found in their average daily time spent using computers and watching television. Early male adolescents generally spent significantly more time than early female adolescents using computers and watching television during the pandemic. The findings on the gender effects were consistent with the preliminary results in our previous study before the pandemic [14] and other studies before [34,35,36] and during the pandemic [37]. Therefore, irrespective of the pandemic, early male adolescents tended to spend more time using display devices than early female adolescents. Regarding their health-related quality of life, early male adolescents, as compared to early female adolescents, reported a significantly lower mean score for three out of the eight scales in the physical and mental domains of health-related quality of life, including physical functioning, role limitation due to physical problems, and social functioning. Though our study did not explore what kinds of recreational activities the early adolescents engaged in when they used different display devices, studies conducted in the U.S.A. [34], Sweden [35], and Australia [36] revealed that male adolescents in different countries spent more time playing video games, resulting in higher screen time than female adolescents. While access to display devices is essential for supporting school-related activities during the lockdown or school closure, it is conceivable that their health-related quality of life is also adversely affected if early male adolescents have more opportunities to use display devices for recreational activities.

The influences of the durations of different display devices on health-related quality of life also differed across gender. Our results suggested that a longer duration of smartphone use was associated with poorer health-related quality of life in three scales of the physical domain, that is, physical functioning, role limitation due to physical problems, and general health; and in all four scales related to the mental domain, that is, vitality (energy/fatigue), social functioning, role limitation due to emotional problems, and mental health (*p* < 0.05), among early female adolescents. In contrast, a longer duration of smartphone, computer, and television use was associated with poorer health-related quality of life in early male adolescents. Specifically, a longer duration of smartphone use was negatively associated with all four scales related to the physical domain and two scales related to the mental domain, that is, the vitality (energy/fatigue) and mental health scales (*p* < 0.05). A longer duration of computer use was negatively associated with one scale related to the physical domain, that is, general health, and one scale related to the mental domain, that is, mental health (*p* < 0.05), whereas a longer duration of watching television was negatively associated with one scale related to the mental domain, that is, role limitation due to emotional problems (*p* < 0.05). Although the durations of smartphone, computer, and television use were not negatively associated with all physical and mental domains of health-related quality of life in early adolescents across gender, the adverse effects of the duration of smartphone use on early adolescents’ health-related quality of life were consistently significant across gender, thus adversely influencing a total of 6 scales of health-related quality of life in males and a total of 7 scales of health-related quality of life in females. Indeed, the negative influence of computer and television use on health-related quality of life was only observed in early male adolescents. Given that early male adolescents spent more time than early female adolescents using computers and watching television, there is a possibility that the differential influence of display devices on health-related quality of life across gender may be due to the difference in the duration of computer and television use. Nevertheless, consistent with our previous study before the pandemic [14], these findings are generally more in line with the displacing social activity hypothesis than the displacing strong ties hypothesis, as proposed by Kraut et al. [20], where a significantly higher amount of time spent using display devices during the pandemic had an adverse influence on early adolescents’ health-related quality of life. Moreover, the results further support the notion that using display devices within a healthy limit can minimize the negative impact on early adolescents’ health-related quality of life and substantiate the importance of investigating the optimal durations of different display devices for an early adolescent to minimize any adverse effects on their health-related quality of life.

Given that early adolescents experience rapid growth and changes during puberty [38], adverse health outcomes associated with their increased screen time during the pandemic—such as lower physical activity and increased sleep problems [18]; blurred vision and eye pain [39]; higher level of depression, anxiety, and inattention [17]; and more significant perceived stress [4]—may have a substantial impact on their normal physical, cognitive, and mental development, leading to public health concerns for this population. Given the potential negative consequences affecting their normal development in the long run, the rising trend of spending more time in front of display devices among early adolescents worldwide during COVID-19 warrants further empirical studies to investigate how such trends impact the public health of this population globally. Moreover, apart from recommendations of limits to screen time from pediatric societies and other associations and evidence-based strategies for parents or caregivers to promote healthy screen hygiene [6,37], it is also essential to consider ways in which policies can facilitate healthy screen use within guidelines and treatments for reducing the adverse impacts of screen time for early adolescents, especially during COVID-19.

This study addressed several limitations of our previous study [14] by examining gender differences in screen time and the influence of screen time on health-related quality of life of early adolescents across gender during COVID-19. Nonetheless, one of the limitations that has not been addressed in this study was the age-related differences and their interaction with gender. Though our study recruited early adolescents with a wide range of ages (9–14 years old), the sample size of each age group still varied, possibly resulting in biased findings if analyzed. A previous study suggested that girls aged 14–15 years had a higher total screen time than girls aged 10–12 years, while an age difference in the total screen time was not found among boys [35]. In addition, older adolescents (13–15 years) also had higher scores for all screen addiction behavior-related statements than those of the younger adolescents (10–12 years) [18]. Therefore, older and younger adolescents may show differences in their screen time and behavior, and the effect of age group in terms of time spent using different display devices, the severity of screen addictive behavior across age or gender, and their influence on health-related quality of life of early adolescents warrant further investigation. Second, this study did not collect the different kinds of activities undertaken by early adolescents using different display devices. Previous studies revealed that girls spent more time using social networking sites [18], while boys reported more than five times more time on video games than girls did [35]. Therefore, apart from the time spent, the activities being performed may also have a differential influence on health-related quality of life. Furthermore, this study was a cross-sectional study, which tested the associations between the influences of the time spent using display devices and early adolescents’ health-related quality of life during COVID-19. The causality and directionality of the results are not conclusive. In addition, as the data collection was conducted through two channels across two years, during which primary and secondary schools partially or entirely transitioned to virtual or online curriculums, depending on the severity of COVID-19 in the community, their use of display devices may vary from time-to-time, and from school-to-school. As the pandemic has become more stabilized or under control in many countries, normal schooling for children and adolescents has gradually resumed worldwide, and more school-based activities can be conducted. The use of display devices for school-related activities may become less prevalent. Therefore, it would be worthwhile to conduct longitudinal studies to capture the causality and/or variation in the influences of these variables throughout different phases of the pandemic.

## 5. Conclusions

This study bridged a knowledge gap by investigating the influence of screen time during COVID-19 on the physical and mental domains of health-related quality of life in early adolescents. While both early male and female adolescents had a comparable amount of time using smartphones on average daily, early male adolescents spent more time using computers and watching television than early female adolescents. A longer duration of smartphone, computer, and television use resulted in significantly poorer physical and mental domains of health-related quality of life among early male adolescents. Health-related quality of life among early female adolescents was negatively affected by a longer duration of smartphone use only. Given that there is a growing body of evidence demonstrating the association between increased screen time and negative consequences for the physical, cognitive, and mental health of children and youth, adverse health outcomes associated with their increased screen time during the pandemic are a public health concern, and the rising trends of spending more time in front of display devices among early adolescents worldwide during COVID-19 warrants further empirical studies to investigate how such trends impact the public health of this population globally.

## Figures and Tables

**Table 1:** Demographic information and descriptive statistics on SF-36 scales.

Variable	Male (*n* = 430)			Female (*n* = 430)			*t*	*p*
	Mean	SD	Range	Mean	SD	Range		
Age (years)	11.526	1.074	9–14	11.472	1.007	9–14	0.753	0.452
SF-36 Scales								
Physical functioning	94.523	13.585	0–00	96.221	9.222	25–100	−2.144	0.032 *
Role limitation due to physical problems	95.116	16.619	0–100	97.151	11.155	0–00	−2.108	0.035 *
Bodily pain	92.793	13.530	0–100	93.758	11.198	41–100	−1.139	0.255
General health	78.574	16.999	10–100	79.177	17.194	25–00	−0.517	0.606
Vitality (energy/fatigue)	72.012	16.682	20–100	73.767	16.974	25–100	−1.530	0.126
Social functioning	91.715	13.113	37.5–100	93.721	11.218	25–100	−2.410	0.016 *
Role limitation due to emotional problems	91.550	23.175	0–100	92.481	20.554	0–100	−0.623	0.534
Mental health	73.405	15.923	16–100	73.461	15.416	16–100	−0.052	0.958

SF-36: 36-item Short Form Health Survey. * *p* < 0.05 (two-tailed).

**Table 2:** Summary of the stepwise multiple linear regressions of the durations of smartphone, computer, and television use as predictors of the physical and mental domains of SF-36 in early male adolescents during COVID-19 (*n* = 430).

Variables	Model 1: Smartphone/Computer/Television Use				Model 2: Smartphone Use			
	*R* ^2^	*F* Value	*p* Value	*β*	*R*^2^∆	*F* Value ∆	*p* Value	*β*
Physical functioning	1.4%	6.038	0.014 *	–1.446 ^†^				
Role limitation due to physical problems	1.2%	5.417	0.020 *	–1.677 ^†^				
Bodily pain	1.4%	5.958	0.015 *	−1.431 ^†^				
General health	1.8%	7.784	0.031 *	−1.783 ^#^	1.0%	4.425	0.036 *	−1.593 ^†^
Vitality (energy/fatigue)	1.6%	6.789	0.009 **	−1.882 ^†^				
Social functioning	1.0%	4.186	0.041 *	−1.013 ^#^				
Role limitation due to emotional problems	1.0%	4.117	0.043 *	−2.258 ^^^				
Mental health	3.4%	14.860	0.000 ***	−2.633 ^†^				

SF-36: 36-item Short Form Health Survey. ^†^ Smartphone, ^#^ Computer, ^^^ Television Use. * *p* < 0.05 (two-tailed). ** *p* < 0.01 (two-tailed). *** *p* < 0.001 (two-tailed).

**Table 3:** Summary of the stepwise multiple linear regressions of the durations of smartphone use as predictors of the physical and mental domains of SF-36 in early female adolescents during COVID-19 (*n* = 430).

Variables	Smartphone Use			
	*R* ^2^	*F* Value	*p* Value	*β*
Physical functioning	1.4%	6.259	0.013 *	−0.996
Role limitation due to physical problems	2.4%	10.417	0.001 **	−1.547
General health	2.6%	11.210	0.000 ***	−2.471
Vitality (energy/fatigue)	4.4%	19.909	0.000 ***	−3.220
Social functioning	1.2%	5.416	0.020 *	−1.128
Role limitation due to emotional problems	1.4%	6.066	0.014 *	−2.186
Mental health	3.4%	15.014	0.000 ***	−2.553

SF-36: 36-item Short Form Health Survey. * *p* < 0.05 (two-tailed). ** *p* < 0.01 (two-tailed). *** *p* < 0.001 (two-tailed).

## Data Availability

The data presented in this study are available upon reasonable request from the corresponding author. The data are not publicly available as they contain information that could compromise the privacy of the early adolescents who participated in the study.
